# Antioxidant Iron Oxide Nanoparticles: Their Biocompatibility and Bioactive Properties

**DOI:** 10.3390/ijms242115901

**Published:** 2023-11-02

**Authors:** Jaewook Lee, Ji-Heon Lee, Seung-Yeul Lee, Sin A Park, Jae Hoon Kim, Dajeong Hwang, Kyung A Kim, Han Sang Kim

**Affiliations:** 1 Research Institute for Biomolecular Chemistry, Dongguk University, Seoul 04620, Republic of Korea; 2 4D Convergence Technology Institute (National Key Technology Institute in University), Korea National University of Transportation, Jungpyeong 27909, Republic of Korea; 3 Genomictree, Inc., 44-6 10-ro Techno, Daejeon 34027, Republic of Korea; 4 Department of Chemical Engineering and Applied Chemistry, Chungnam National University, Daejeon 34134, Republic of Korea; 5 Yonsei Cancer Center, Seoul 30722, Republic of Koreamodeerfhs@yuhs.ac (H.S.K.); 6 Division of Medical Oncology, Department of Internal Medicine, Graduate School of Medical Science Brain Korea 21 Project, Yonsei University College of Medicine, Seoul 03722, Republic of Korea

**Keywords:** iron oxide, antioxidant iron oxide, gallic acid, biocompatibility, antioxidant effect, nanomaterials, cell protective effect

## Abstract

A lot of nanomaterials have been applied to various nano-biotechnological fields, such as contrast agents, drug or gene delivery systems, cosmetics, and so on. Despite the expanding usage of nanomaterials, concerns persist regarding their potential toxicity. To address this issue, many scientists have tried to develop biocompatible nanomaterials containing phytochemicals as a promising solution. In this study, we synthesized biocompatible nanomaterials by using gallic acid (GA), which is a phytochemical, and coating it onto the surface of iron oxide nanoparticles (IONPs). Importantly, the GA-modified iron oxide nanoparticles (GA-IONPs) were successfully prepared through environmentally friendly methods, avoiding the use of harmful reagents and extreme conditions. The presence of GA on the surface of IONPs improved their stability and bioactive properties. In addition, cell viability assays proved that GA-IONPs possessed excellent biocompatibility in human dermal papilla cells (HDPCs). Additionally, GA-IONPs showed antioxidant activity, which reduced intracellular reactive oxygen species (ROS) levels in an oxidative stress model induced by hydrogen peroxide (H_2_O_2_). To investigate the impact of GA-IONPs on exosome secretions from oxidative stress-induced cells, we analyzed the number and characteristics of exosomes in the culture media of HDPCs after H_2_O_2_ stimulation or GA-IONP treatment. Our analysis revealed that both the number and proportions of tetraspanins (CD9, CD81, and CD63) in exosomes were similar in the control group and the GA-IONP-treated groups. In contrast, exosome secretion was increased, and the proportion of tetraspanin was changed in the H_2_O_2_-treated group compared to the control group. It demonstrated that treatment with GA-IONPs effectively attenuated exosome secretion induced by H_2_O_2_-induced oxidative stress. Therefore, this GA-IONP exhibited outstanding promise for applications in the field of nanobiotechnology.

## 1. Introduction

In recent decades, nanomaterials have played significant roles in the field of nanobiotechnology, such as drug delivery systems, gene delivery carriers, imaging agents, and so on [1,2,3,4,5,6]. However, several significant concerns have emerged related to biocompatibility, toxicity, and the stability of nanoparticles, which means it potentially restricts their suitability for applications in the field of nanobiotechnology [7,8,9,10]. To address these issues and enhance biocompatibility, natural substances have been employed for the preparation of nanomaterials and were suggested as an alternative strategy.

Among the natural products, phytochemicals have been modified on the surface of nanomaterials to be utilized as the surface stabilizer and active material. The significant characteristics of phytochemicals are chemical diversity, biological properties, and low toxicity, and it was reported that the combinations of phytochemicals and nanomaterials have been demonstrated to enhance biological activity, biocompatibility, and low toxicity [11,12,13]. Gallic acid (GA), a phytochemical, has excellent potential for antioxidant effects, antiinflammation, and anticancer effects [14,15,16,17,18]. It has exhibited the capability to scavenge free radicals and, through this reactivity, it could protect cells and tissues from oxidative stress [14]. Due to this excellent bioactivity, GA has been considered a potential candidate for functional material on the surface of a nanostructure [19].

On the other hand, it is widely recognized that reactive oxygen species (ROS)-induced oxidative stress generated by H_2_O_2_ can destroy the balance within cellular systems, ultimately leading to cell death. To reduce an excess of ROS, cells utilize a variety of regulatory mechanisms to maintain homeostasis. Even exposure to a low concentration of H_2_O_2_ can stimulate the secretion of exosomes in different cell types, such as lens epithelial cells (LECs), HEK293 cells, and leukemia/lymphoma T and B cells [20,21,22]. Moreover, recent studies have shown that exosomes derived from healthy cells effectively alleviate oxidative stress in recipient cells through various mechanisms, both in vitro and in vivo [23,24,25,26]. In other words, exosomes serve a crucial function as messengers between cells, influencing and controlling various cellular processes [27,28,29]. Furthermore, tetraspanins on the exosome are small transmembrane proteins, and they are expressed in various types, such as CD9, CD81, CD63, and so on. These tetraspanins have the potential to impact a range of biological functions, such as adhesion, activation, mobility, proliferation, and more [30,31]. In addition, they could play a role as biomarkers due to their portion on the exosome, showing different tendencies depending on the cell type [32,33]. Exosomes have played an important role in maintaining homeostasis under oxidative stress. Hence, we hypothesized that biocompatible nanomaterial that possessed antioxidant abilities could protect cells and maintain homeostasis against oxidative stress and relate with exosome secretion.

In this study, we prepared GA-functionalized iron oxide nanoparticles (GA-IONPs) as antioxidant and biocompatible nanomaterials for potential applications in the nano-bio fields. Initially, the cytotoxicity of GA-IONPs was characterized depending on the various concentrations up to 0.5 mg/mL. Subsequently, the antioxidant activity and cell protective effect of GA-IONPs against oxidative damage were evaluated via in vitro study with H_2_O_2_ stimulation in human dermal papilla cells (HDPCs). Also, the exosome secretion was monitored depending on the H_2_O_2_-induced oxidative stress and GA-IONP treatment in HDPC.

Our findings proved that GA-IONP was a non-toxic nanomaterial possessing strong antioxidant properties. Also, the protective effect of GA-IONP was confirmed through the observation of tetraspanin tendency in released exosomes from H_2_O_2_-stimulated and GA-IONP-treated HDPCs; the results showed outstanding cell protection behavior. Consequently, GA-IONPs have promising potential for use in nano-bio medical applications.

## 2. Results

**Figure 1:**
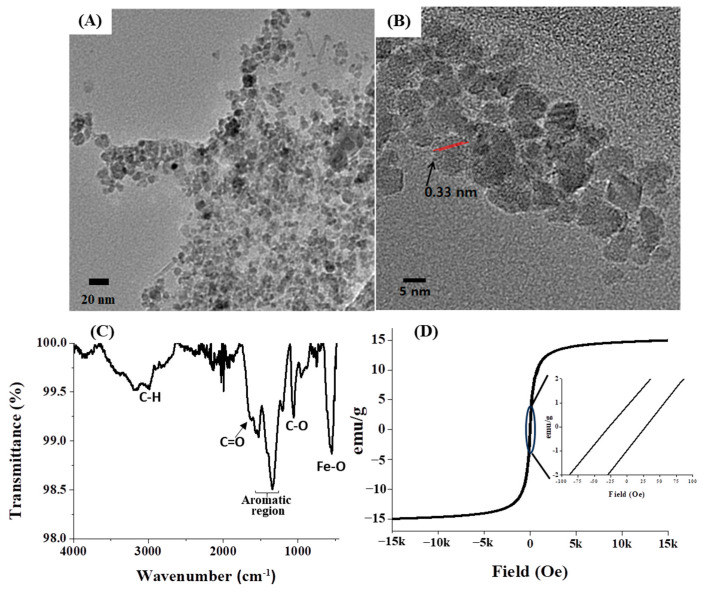
The morphology and size observations. (**A**) TEM image, (**B**) HR-TEM image, (**C**) FT-IR spectrum for functional group analysis, and (**D**) SQUID analysis for magnetic property of GA-IONP.

Subsequent to the preparation of GA-IONPs, their physicochemical properties were characterized by various methods. Firstly, their morphology and structure were observed by using high-resolution transmission electron microscopy (HR-TEM), as depicted in Figure 1A,B. The particles displayed an approximate diameter of 9.8 nm and a spherical shape. Notably, the GA-IONPs were uniformly dispersed without aggregation, which can be attributed to the surface modification with the organic acid, GA. Typically, carboxylic groups in organic acid can bind to the surface of IONP, so in this case, the carboxylic group on GA was able to attach to the IONP surface, preventing aggregation between the IONPs. Secondly, the functional groups of GA-IONPs were characterized by using Fourier-transform-infrared (FT-IR) spectroscopy. According to the spectrum in Figure 1C, the vibration mode of Fe-O was measured around 600 cm^−1^, which was also observed in the IONP sample (Figure S1A). The C=C vibration in the aromatic group of GA on the IONP was identified between 1400 cm^−1^ and 1600 cm^−1^. The stretching mode of the carboxylic group of GA appeared at 1680 cm^−1^. In this case, due to the attachment of the carboxylic group of GA onto the IONP, C=O vibration energy might be shifted more than that of pure GA. The C-O stretching mode was analyzed at about 1019 cm^−1^; these results revealed that GA was successfully modified on the surface of IONP. Additionally, O-H vibrations from trihydroxyl groups of GA on the surface of IONP were shown around 3300~3500 cm^−1^, and it was also measured in the pure GA (Figure S1B). The magnetic property of GA-IONP was evaluated by using a superconducting quantum interference device (SQUID) measurement at room temperature between −15 kOe and 15 kOe (Figure 1D). The magnetic hysteresis relationship exhibited a non-linear shape, and a hysteresis loop was shown. The inserted magnetic hysteresis curve indicated a remanence effect measured at about 0.88 and −0.96 emu/g, and coercive force was indicated at approximately 28.01 and −27.07 Oe. Furthermore, the zeta potential of GA-IONP was measured, demonstrating a significantly negative charge of about −36.7 mV (Figure S2).

**Figure 2:**
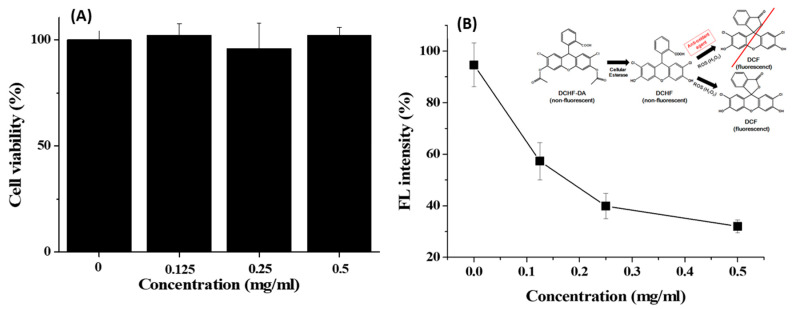
(**A**) Cell viability of GA-IONPs and (**B**) FL intensity (%) measurement after H_2_O_2_ treatment depending on the various concentrations of GA-IONPs for antioxidant effect test by DCHF-DA assay.

The cytotoxicity of GA-IONP was determined in the human dermal papilla cells (HDPC) at various concentrations through MTT assay for a 24 h period. As shown in Figure 2A, the cell viability remained above 95% even when exposed to concentrations as high as 0.5 mg/mL. This outcome indicated that GA-IONP exhibited no cytotoxicity and, therefore, could be effectively utilized in nanobiotechnology applications. Furthermore, its antioxidant effect was evaluated with HDPC using a DCF assay kit against H_2_O_2_ as an inducer of ROS. In general, DCFH-DA is converted to DCFH by cellular esterases in the cell, and if DCFH is subsequently oxidized by ROS, it is transformed into DCF, which emits a robust green fluorescence. This means that the fluorescence intensity of DCF varies depending on the antioxidant activity of the material against ROS, allowing for the estimation of its antioxidant effectiveness. In Figure 2B, when HDPC was only stimulated by H_2_O_2_, high fluorescent (FL) intensity was measured, and it was thought that almost all DCFH might be converted to DCF in the cell due to the activity of ROS. However, as the concentration of the GA-IONPs increased, the FL intensity decreased by approximately 70% at a concentration of 0.5 mg/mL. It meant that GA-IONPs had the function of antioxidants and acted as ROS scavengers, so they could inhibit the activity of H_2_O_2_. As a result, it was not allowed to transform from DCHF to DCF in the cell cytosol.

**Figure 3:**
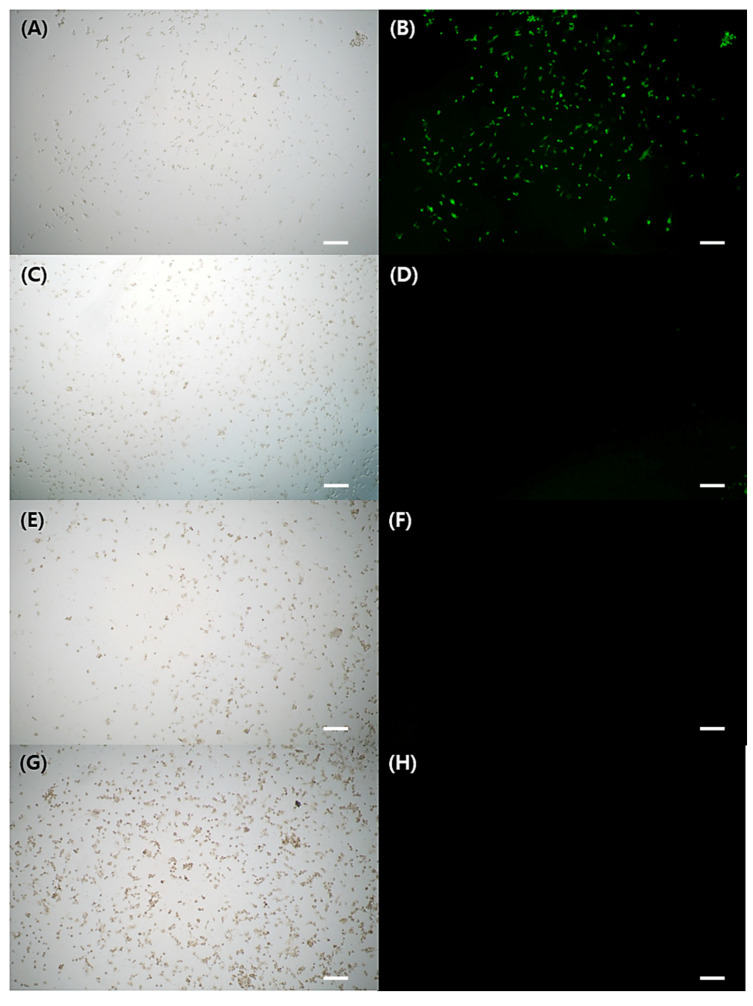
The cytoprotective properties against H_2_O_2_ stimulation. Observation of FL microscope of (**A**,**B**) without treatment of GA-IONP, (**C**,**D**) 0.125 mg/mL, (**E**,**F**) 0.25 mg/mL, and (**G**,**H**) 0.5 mg/mL of GA-IONP. The scale bar indicates 100 μm.

Figure 3A–H depicted FL microscope images of cells exposed to either H_2_O_2_ stimulation alone or H_2_O_2_ treatment with GA-IONPs. In Figure 3B, green FL images were shown in the cells by the presence of DCF, which was converted from DCHF through H_2_O_2_ activity. However, in the H_2_O_2_/GA-IONP-treated group (Figure 3C–H), only a small number of cells exhibited green FL images. These results were consistent with the FL intensity measurement result in Figure 2B. Thus, it proved that these GA-IONPs could protect the cells against oxidative stress and possess a strong antioxidant effect.

**Figure 4:**
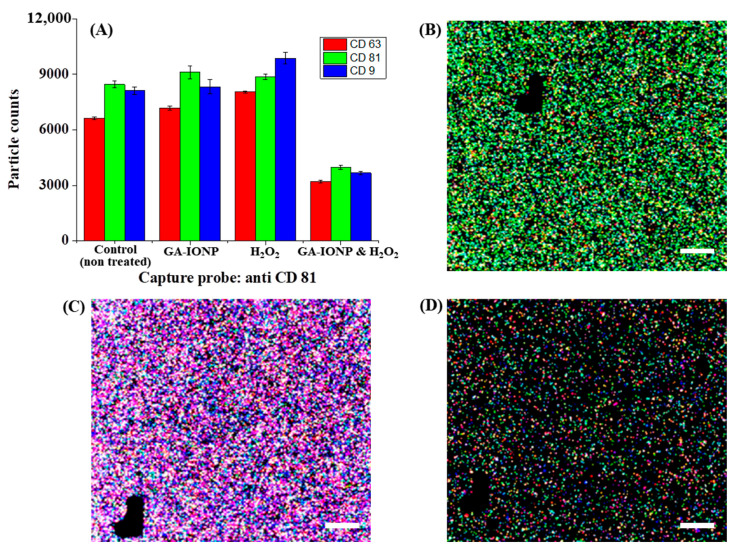
Tetraspanin characterization of HDPC-derived exosomes after H_2_O_2_ or H_2_O_2_/GA-IONP treatment on anti-CD81 capture spots. (**A**) Number and ratio of dye-tetraspanin-labeled exosomes and FL images of (**B**) control (non-treated), (**C**) H_2_O_2_-stimulated group, and (**D**) H_2_O_2_/GA-IONP treated group. The scale bar was 20 μm.

**Figure 5:**
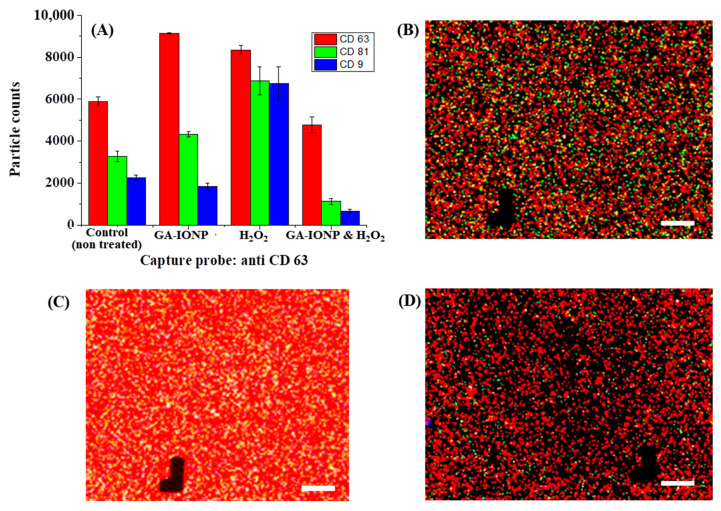
Tetraspanin characterization of HDPC-derived exosomes after H_2_O_2_ or H_2_O_2_/GA-IONP treatment on anti-CD63 capture spots. (**A**) Number and ratio of dye-tetraspanin-labeled exosomes and FL images of (**B**) control (non-treated), (**C**) H_2_O_2_-stimulated group, and (**D**) H_2_O_2_/GA-IONP-treated group. The scale bar was 20 μm.

In this study, we also conducted an analysis of the number and proportion of exosomes based on the expression of tetraspanin after ROS stimulation by using the exosome analysis platform, Exoview R100, and a standard tetraspanin kit. The exosomes secreted from cells treated with GA-IONP, H_2_O_2_, or H_2_O_2_/GA-IONP were dropped onto the exoview tetraspanin-sensing chip containing tetraspanin antibodies (CD63, CD81, and CD9) and MIgG (negative control)-immobilized capture spots and incubated. During the incubation step, it was possible to capture and deposit the exosomes on the sensing chip. After that, the exosomes on each capture spot were labeled with dye-conjugated tetraspanin Ab (AF647-CD63 (red), AF555-CD81 (green), and AF488-CD9 (blue)) [34]. This labeling allowed us to quantify the particle counts and analyze the colocalization tendency of the exosomes. The black marks visible in the images functioned as address patterns (in Figure 4 and Figure 5). This exosome measurement equipment is automatically operated, and for spot identification and signal reading, address patterns are necessary on the sensing spots [35].

Figure 4 explains the numbers and tetraspanin ratio of HDPC-derived exosomes on the anti-CD81 capture spots after GA-IONP, H_2_O_2_, or H_2_O_2_/GA-IONP treatment. As depicted in Figure 4A, in the cases of control, GA-IONP treated group, and H_2_O_2_/GA-IONP treated group, the highest exosome count was shown under AF555-CD81-labeled conditions (green column). In contrast, in the group treated solely with H_2_O_2_, a different trend was indicated in that the highest exosome count was measured under the AF488-CD9-labeled condition (blue column). In addition, the pattern of the exosome count was visualized depending on the labeling condition in Figure 4B–D. In the case of control and H_2_O_2_/GA-IONP, numerous green dots were predominantly visible, indicating a high presence of AF555-CD81-labeled exosomes in the samples (Figure 4B,D). A similar pattern with a high number of green spots was also observed in the only GA-IONP treatment group (Figure S3). In contrast, many blue and purple (coexistence of red and/or blue labeled exosome) dots were exhibited in the H_2_O_2_ stimulated group, so it was estimated that AF488-CD9-labeled exosomes were highly occupied in the sample (Figure 4C). These FL images were well matched with the column chart in Figure 4A.

A similar phenomenon was observed in CD63 capture spots, as illustrated in Figure 5. In this case, the same distribution patterns of exosome counts labeled with different dye-tetraspanins were observed in the control group, the GA-IONP-treated group, and the H_2_O_2_/GA-IONP-treated group (Figure 5A). However, a dissimilar pattern was observed in the H_2_O_2_-treated group, which is that the ratio of AF555-CD81 and AF488-CD9-labeled exosomes was almost the same, and both labeled exosomes were slightly lower than that of AF647-CD63-labeled exosomes. Additionally, the FL images exhibited the exosomes labeled with AF647-CD63, AF555-CD81, and AF488-CD9 in Figure 5B–D and Figure S4. Comparing Figure 5B,D and Figure S4 to Figure 5C, a high abundance of white spots (the coexistence of green, blue, and red) was observed in Figure 5C, indicating a different color pattern. The result of these figures was also well-matched with the distribution patterns of the labeled exosomes (Figure 5A).

It has been well established that the expression of CD9 and CD81 in the exosome was closely related to oxidative stress, owing to their functional roles [36]. Furthermore, it was reported that among the tetraspanin proteins, CD9 and CD81 are closely associated with cellular processes such as cell proliferation and inflammation. Both proteins can mitigate senescence and inflammation, partially by upholding the expression of SIRT1 [37].

The results from the analysis of tetraspanins in secreted exosomes have provided strong evidence of a robust connection between oxidative stress and the tetraspanin composition of exosomes. GA-IONPs could sustain this tetraspanin ratio against oxidative stress. Therefore, it proved that GA-IONPs possessed the ability to protect cells and preserve homeostasis when exposed to ROS-induced stimulation, aligning with our initial expectations.

## 3. Discussion

GA is a polyphenolic compound that can be obtained from natural products such as chestnuts, acorns, gallnuts, and so on. The reduction potential of GA was previously estimated to be 0.863 V, which was sufficient to reduce Au^3+^ to Au^0^ and create the Au NPs [38]. This indicated that GA possessed potent antioxidant properties due to its high reduction potential. Also, in the previous study, GA-IONP could convert the Au^3+^ to AuNPs on the graphene and CNT, and as a result, GA-IONP/AuNP binary NP-modified graphene and CNT were successfully prepared. In that study, it was utilized as a reducing agent and surface modification material [27]. Additionally, the stability of GA-IONP has been explored in earlier studies. The NP maintained its reduction activity and exhibited no precipitation even after being stored for over 1 month in a refrigerator, so it was enough to use as a reducing agent to prepare the GA-IONP/metal NP-decorated graphene or CNT [27,39]. So, it suggested that this NP possessed good stability. On the other hand, in this study, the biocompatibility of GA-IONP was characterized, and this NP was synthesized using eco-friendly methods without harsh conditions or high temperatures, and they were well dispersed in the DI water [27,39]. The high dispersity of GA-IONPs can be attributed to the presence of trihydroxyl groups from GA on the surface of the IONP. These OH groups could generate substantial charges at the surface, leading to strong repulsive forces that prevent the aggregation of IONPs [27,39]. Furthermore, according to the previous study, the relative quantity of GA in the GA-IONP nanocomposite was estimated to be approximately 15% using thermogravimetric analysis (TGA) [40].

According to the results from the cytotoxicity assay of GA-IONPs, cell viability remained consistent in all tested concentrations, ranging from 0 to 0.5 mg/mL, and remained around 95% after the 24 h treatment period. So, it was confirmed that this GA-IONP possessed high biocompatibility. In a prior study, no cytotoxicity was observed, even at a concentration of 1000 ppm for bare-IONP [41]. Furthermore, we did not recognize any genotoxic effects associated with IONPs [41], which suggests that GA-IONPs prepared from natural products, like GA, were unlikely to exhibit genotoxicity as well.

The antioxidant effect of GA-IONPs was evaluated depending on the various concentrations under H_2_O_2_ stimulation. The presence of GA-IONPs in the cell culture environments seemed to inhibit the activity of H_2_O_2_, consequently resulting in a prevented conversion of DCHF to DCF in the cytoplasm, leading to low green FL intensity. This effect might be caused by the functionality of IONPs with GA on the surface. On the other hand, in the case of sole H_2_O_2_ stimulation conditions, the high intensity of green FL was measured, and green fluorescence was observed within the cell, which meant that GA-IONP could protect the cell against ROS-induced oxidative stress.

Furthermore, we conducted an analysis of HDPC-derived exosome formation and characteristics following treatment with GA-IONP, H_2_O_2_, and H_2_O_2_/GA-IONP. Interestingly, in the groups of control and GA-IONP and H_2_O_2_/GA-IONP treatment, the distribution ratio of both colocalization and quantity of exosomes labeled with tetraspanins AF555-CD81 and AF488-CD9 were nearly the same at the anti-CD81 and anti-CD63 capture spots on the exosome tetraspanin analysis chips. However, in the case of the H_2_O_2_ stimulation condition, both AF488-CD9 and AF555-CD81 levels were increased, and different tendencies were exhibited. It is well established that the expression of CD9 and CD81 can be modulated by inflammation [36,37]. In other words, in response to oxidative stress induced by H2O2, cells attempted to protect themselves by increasing the levels of both tetraspanin proteins. Consequently, the exosomes released in only H_2_O_2_-stimulated cells had a higher proportion of CD9 and CD81 tetraspanins. However, GA-IONPs could effectively prevent H_2_O_2_ activity and subsequently reduce cell damage against oxidative stress; the proportion of tetraspanins in the H_2_O_2_/GA-IONP treatment group closely resembled that of the control group. Moreover, the trend observed in the non-control group and GA-IONP-treated group was quite similar, suggesting that toxicity might not be a significant concern. Nevertheless, a more detailed study of the mechanisms and pathways involved in oxidative stress and cell protection processes will be presented in future studies.

## 4. Materials and Methods

### 4.1. Materials and Instruments

Gallic acid (GA; 3,4,5-trihydroxy benzoic acid), FeCl_3_, FeCl_2_·4H_2_O, and 25% NH_4_OH solution were purchased from Sigma-Aldrich (Yongin, Republic of Korea). The reactive oxygen species (ROS) assay kit, which includes the cell-permeable fluorogenic probe 2’,7’-dichlorodihydrofluorescein diacetate (DCFH-DA), was also bought from Sigma-Aldrich (Yongin, Republic of Korea) to evaluate the H_2_O_2_-scavenging activity of GA-IONPs. The human follicle dermal papilla cells (HDPC) were purchased from the Promo cell (Heidelberg, Germany), and the follicle dermal papilla cell growth medium kit was obtained from Promo cell (Heidelberg, Germany). The 1% penicillin–streptomycin was bought from Gibco (New York, NY, USA). MTT (3-[4,5-dimethylthiazol-2-yl]-2,5-diphenyltetrazolium bromide) assay kit was purchased from Abcam (Cambridge, UK). ExoView Tetraspanin Kits containing tetraspanin antibody dye (AF488-anti CD9 and AF555-anti CD81) were obtained from NanoView Biosciences (Boston, MI, USA). The functional groups of hybrid structures were monitored by FT-IR spectroscopy (Cary 610, Agilent Technologies, Santa Clara, CA, USA). The magnetic property of particles was analyzed by the Magnetic Property Measurement System (MPMS3-Evercool (SQUID), quantum design, USA). The morphologies and size of the NPs were observed by HR-TEM at 200 kV (JEM-2100F, JEOL, Tokyo, Japan). The FL intensity and FL image of H_2_O_2_ or GA-IONP and H_2_O_2_-treated cells were observed via a microplate reader (Bio Tek Synergy HTX, USA) and FL microscopy (IX73, Olympus, Japan), respectively. The zeta potential of GA-IONP was measured by Nano ZS zetasizer (Malvern Instruments, Worcestershire, UK).

### 4.2. Synthesis of GA-IONPs

GA-IONPs were prepared via a coprecipitation process. Firstly, FeCl_3_ (1 mmol, 0.1622 g) and FeCl_2_·4H_2_O (0.5 mmol, 0.0994 g) were dissolved in deionized (DI) water (20 mL). Then, 25% NH_4_OH solution (0.6 mL) was added to the ferric and ferrous chloride mixture and stirred at 500 rpm for 10 min to form the IONP. Subsequently, GA (1.5 mmol, 0.255 g) powder was poured into the black IONP solution and continuously stirred at 90 °C for 30 min. Owing to the binding between GA and iron in the GA-IONP structure, the solution color changed from black to deep violet. After stirring, GA-IONP was purified and precipitated with excess acetone and separated by an external magnetic force using a commercial neodymium magnet (1.4 T) and dried in the vacuum oven (OV11, JEIO TECH, Daejeon, Republic of Korea).

### 4.3. Biological Activity Test of GA-IONP

To characterize the biological activities of GA-IONP, such as cytotoxicity and antioxidant effect, human follicle dermal papilla cells (HDPC) were cultured in follicle dermal papilla cell growth medium from a growth medium kit containing 1% penicillin–streptomycin at 37 °C, 5% CO_2_ until they reached 90% confluence. Firstly, the cytotoxicity of GA-IONP was characterized via MTT assay as follows. Before the GA-IONP treatment to the cells, the NP sample was gently mixed using a micropipette and added. To improve the dispersity of the GA-IONP, short-term sonication (1–5 min) was conducted before the treatment. This NP is stable and well dispersed; the aggregation and precipitation were not observed even after sonication. Briefly, HDPC was treated by increasing the concentration of GA-IONP from 0 mg/mL to 0.5 mg/mL and then incubated with MTT for the last 4 h in the culture period (24 h) [42,43,44]. After that, the absorbance was recorded at 570 nm by using a microplate reader to determine the relative cell viability.

Secondly, the antioxidant effect of GA-IONP was evaluated against H_2_O_2_ by using a reactive oxygen species (ROS) assay kit containing a cell-permeable fluorogenic probe 2’,7’-dichlorodihydro-fluorescein diacetate (DCFH-DA). In this test, various concentrations of GA-IONPs (0 mg/mL~0.5 mg/mL) were treated with HDPC cells and then incubated for 24 h. After that, 0.9 mM of H_2_O_2_ was introduced to GA-IONP-treated HDPC for 30 min. Subsequently, 10 μM of DCHF-DA was added and allowed to incubate in the dark for an additional 30 min. The FL intensity of DCF that was converted from DCHF-DA by H_2_O_2_ was analyzed by using a microplate reader at an excitation wavelength of 480 nm and an emission wavelength of 530 nm to evaluate the antioxidant properties of GA-IONP. Furthermore, the FL images of the above sample were observed using an FL microscope.

### 4.4. Exosome Analysis Depending on the ROS and GA-IONP Treatment

The tetraspanin ratio and numbers of exosomes from GA-IONPs/H_2_O_2_-treated HDPC or H_2_O_2_-stimulated HDPC were characterized by using the Exoview platform and exoview tetraspanin kits. To perform this test, firstly, the culture media after GA-IONPs, GA-IONPs/H_2_O_2_ treatment, or H_2_O_2_ stimulation to the HDPC were collected and centrifuged under 1500 rpm for 10 min to remove the cell debris and large impurities. Then, each supernatant was used as the sample for the characterization of exosomes, and 35 μl of each solution was dropped onto the Exoview tetraspanin chip that was composed of sensing spots, including anti-CD81, anti-CD63, anti-CD9, and negative control (mouse IgG) and incubated overnight. According to the Exoview analysis protocol, this sensing chip possesses tetraspanin antibodies so that they can capture and recognize the tetraspanin on the exosomes without a precise separation process. This meant it was enough to isolate the exosome at low speed for the elimination of impurities. After overnight incubation, the chip that captured exosomes was washed with washing buffer, and the FL dye-conjugated anti-tetraspanins (AF647-CD63 (red), AF555-CD81 (green), and AF488-CD9 (blue)) were labeled and measured to confirm the counts and colocalization of exosomes by using the Exoview R100 platform.

## 5. Conclusions

In this research, we successfully synthesized biocompatible GA-IONPs through a mild process in deionized water. These NPs demonstrated minimal cytotoxicity, and the cell viability remained above 95% even at a high concentration of approximately 0.5 mg/mL. In addition, remarkable biological properties were exhibited, including antioxidant effects and cell protection capabilities against ROS, particularly H_2_O_2_. The ROS activity was significantly reduced by 70% when treated with 0.5 mg/mL of GA-IONP. In particular, we conducted an analysis of the tetraspanin composition in exosomes released from HDPCs treated with GA-IONPs and GA-IONPs/H_2_O_2_, as well as those stimulated solely with H_2_O_2_. In this case, the portion of CD63, CD81, and CD9 in the GA-IONP-treated group was similar to the control group, but the H_2_O_2_ treatment group was totally different. The results proved the ability of GA-IONPs to effectively mitigate oxidative stress in cells induced by ROS. Consequently, this study showed the possibility of the potential of GA-IONPs for applications in the field of nanobiotechnology. Furthermore, GA-IONP exhibits synergic properties, including antioxidant and cell protection properties and magnetic characteristics, making it suitable for use as a theranostic agent. Further in vivo studies will be conducted to explore their utility in drug delivery systems and other related applications.

## Data Availability

The data presented in this study are available on request from the corresponding author.

## Supplementary Materials

The following supporting information can be downloaded at: https://www.mdpi.com/article/10.3390/ijms242115901/s1.
