# Supplementary material for: Antioxidant Iron Oxide Nanoparticles: Their Biocompatibility and Bioactive Properties
Source: Int J Mol Sci. 2023 Nov 2;24(21):15901. doi: 10.3390/ijms242115901 (PMC10649306; doi:10.3390/ijms242115901)
Supplement: Supplementary file 1 [file ijms-24-15901-s001.zip › ijms-2673508-supplementary.pdf]

Supplementary Information

Antioxidant Iron Oxide Nanoparticles: Their Biocompatibility and Bioactive Properties

Jaewook Lee [1,*], Ji-Heon Lee [2], Seung-Yeul Lee [3], Sin A Park [3], Jae Hoon Kim [3], Dajeong Hwang [4], Kyung A Kim [5] and Han Sang Kim [5,6]

1 Research Institute for Biomolecular Chemistry, Dongguk University, Seoul 04620, Republic of Korea
2 4D Convergence Technology Institute (National Key Technology Institute in University), Korea National University of Transportation, Jungpyeong 27909, Republic of Korea
3 Genomictree, Inc., 44-6 10-ro Techno, Daejeon 34027, Republic of Korea
4 Department of Chemical Engineering and Applied Chemistry, Chungnam National University, Daejeon 34134, Republic of Korea
5 Yonsei Cancer Center, Seoul 30722, Republic of Korea; modeerfhs@yuhs.ac (H.S.K.)
6 Division of Medical Oncology, Department of Internal Medicine, Graduate School of Medical Science Brain Korea 21 Project, Yonsei University College of Medicine, Seoul 03722, Republic of Korea
* Correspondence: loveu@dongguk.edu

[Supplementary images]

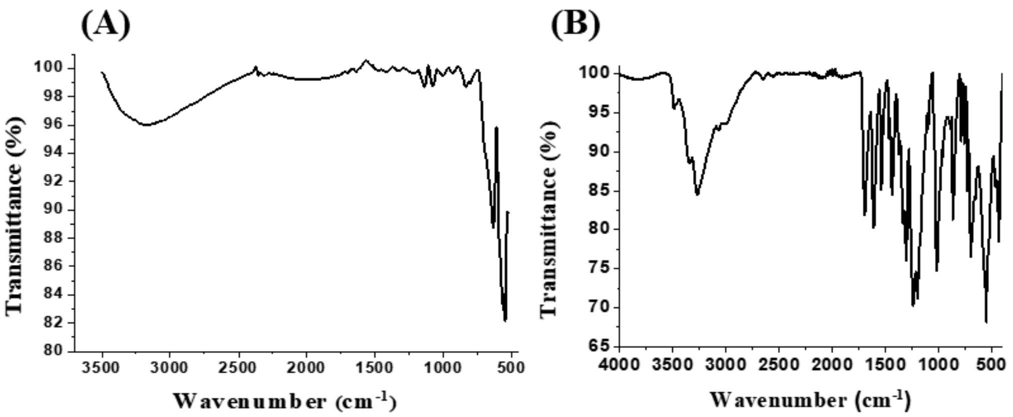

Figure S1. FT-IR spectrum of (A) IONP and (B) GA

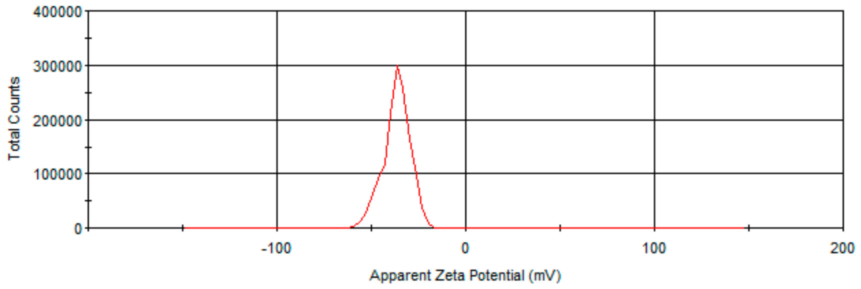

Figure S2. Zetapotential of GA-IONP

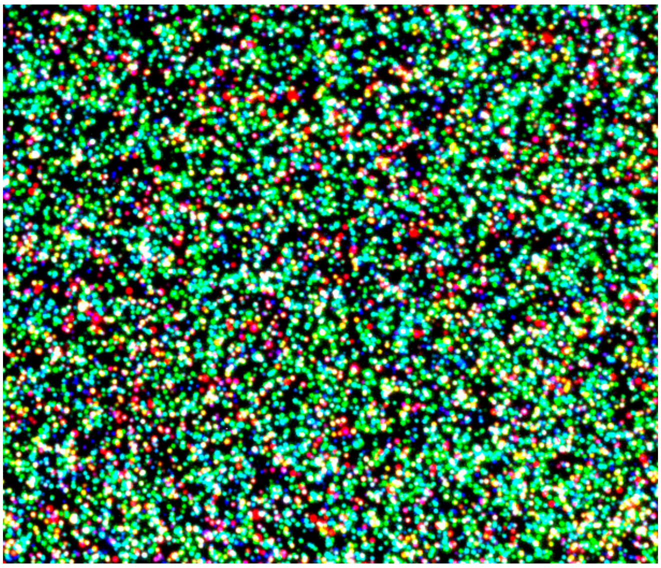

Figure S3. FL image of released exosomes in GA-IONP treatment condition at CD81 capture spot.

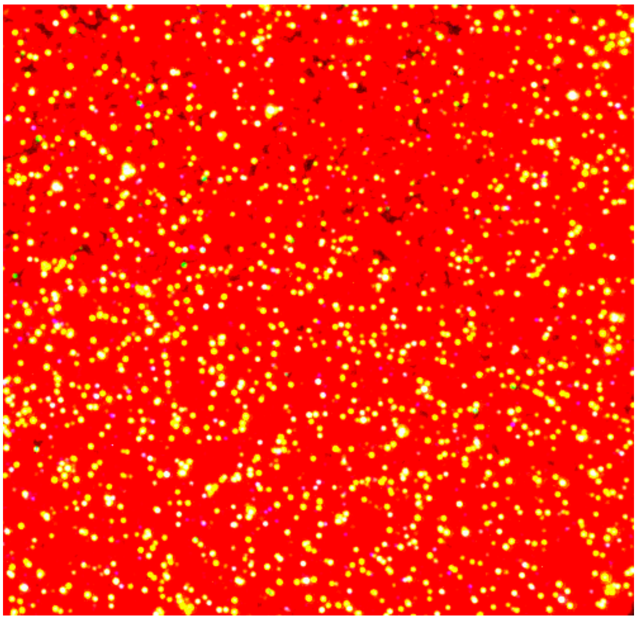

Figure S4. FL image of released exosomes in GA-IONP treatment condition at CD63 capture spot.
